# Perceptual inference employs intrinsic alpha frequency to resolve perceptual ambiguity

**DOI:** 10.1371/journal.pbio.3000025

**Published:** 2019-03-13

**Authors:** Lu Shen, Biao Han, Lihan Chen, Qi Chen

**Affiliations:** 1 Center for Studies of Psychological Application and School of Psychology, South China Normal University, Guangzhou, China; 2 Guangdong Key Laboratory of Mental Health and Cognitive Science, South China Normal University, Guangzhou, China; 3 Donders Institute for Brain, Cognition and Behaviour, Radboud University, Nijmegen, The Netherlands; 4 School of Psychological and Cognitive Sciences and Beijing Key Laboratory of Behavior and Mental Health, Peking University, Beijing, China; Newcastle University Medical School, UNITED KINGDOM

## Abstract

The brain uses its intrinsic dynamics to actively predict observed sensory inputs, especially under perceptual ambiguity. However, it remains unclear how this inference process is neurally implemented in biasing perception of ambiguous inputs towards the predicted percepts. The process of perceptual inference can be well illustrated by the phenomenon of bistable apparent motion in the Ternus display, in which subjective perception spontaneously alternates between element motion (EM) and group motion (GM) percepts depending on whether two consecutively presented frames are grouped over time or not. The frequency of alpha-band oscillations has long been hypothesized to gate the temporal window of perceptual grouping over time. Under this hypothesis, variation in the intrinsic alpha frequency should predict perceptual outcome of the bistable Ternus display. Moreover, we hypothesize that the perception system employs this prior knowledge on intrinsic alpha frequency to resolve perceptual ambiguity, by shifting perceptual inference towards the predicted percepts. Using electroencephalography and intracranial recordings, we showed that both between and within subjects, lower prestimulus alpha frequencies (PAFs) predicted the EM percepts since the two frames fell in the same alpha cycle and got temporally integrated, while higher PAFs predicted the GM percepts since the two frames fell in different alpha cycles. Multivariate decoding analysis between the EM percepts with lower PAFs and the GM percepts with higher PAFs further revealed a representation of the subsequently reported bistable percept in the neural signals shortly before the actual appearance of the second frame. Therefore, perceptual inference, based on variation in intrinsic PAFs, biases poststimulus neural representations by inducing preactivation of the predicted percepts. In addition, enhanced prestimulus blood-oxygen-level–dependent (BOLD) signals and network dynamics in the frontoparietal network, together with reduced prestimulus alpha power, upon perceiving the EM percepts suggest that temporal grouping is an attention-demanding process.

## Abstract 2

Electroencephalography, intracranial recording, and fMRI measurements of the human brain reveal how perceptual inference employs intrinsic brain activity to bias the perception of ambiguous sensory information towards predicted percepts.

## Author summary

Our subjective perception of the external world is constantly shaped not only by sensory inputs but also by the real-time internal status of our own brain. Upon facing an ambiguous sensory input, our brain employs its intrinsic dynamics to bias perception towards the predicted percept, resolving in this manner perceptual ambiguity. However, it remains poorly understood how this process is implemented in the brain. Using electroencephalogram and functional MRI in healthy participants and intracranial recordings in epileptic patients, we show that neural rhythms in the alpha frequency (8–13 Hz)—one of the predominant neural oscillations in the visual system—gate the time window for perceptual grouping: two consecutively presented frames in a constant ambiguous dynamic visual scene will be temporally integrated if they fall in the same alpha cycle but spatially integrated if they fall in different alpha cycles. Moreover, our brain employs the real-time speed of its intrinsic alpha oscillations to actively predict the most possibly observed percepts by inducing preactivation of neural representations that resemble the one evoked by the actual stimuli, even before it is presented.

## Introduction

The brain is increasingly being understood as engaged in probabilistic unconscious perceptual inference to actively predict and explain observed sensory inputs, which helps to resolve ambiguity in sensory signals [1–6]. Therefore, our perception of the world is not simply based on our sensory inputs. Instead, what we perceive is heavily altered by contextual information [7–10] and expectations [11–14]. Besides context and expectation, intrinsic neural oscillatory signatures and organization status of the brain dramatically modulate the perceptual outcome of ambiguous stimuli [15–19]. However, it remains unclear how perceptual inference employs intrinsic brain activity to bias the perception of ambiguous sensory information towards predicted percepts with the progress of time.

**Fig 1:**
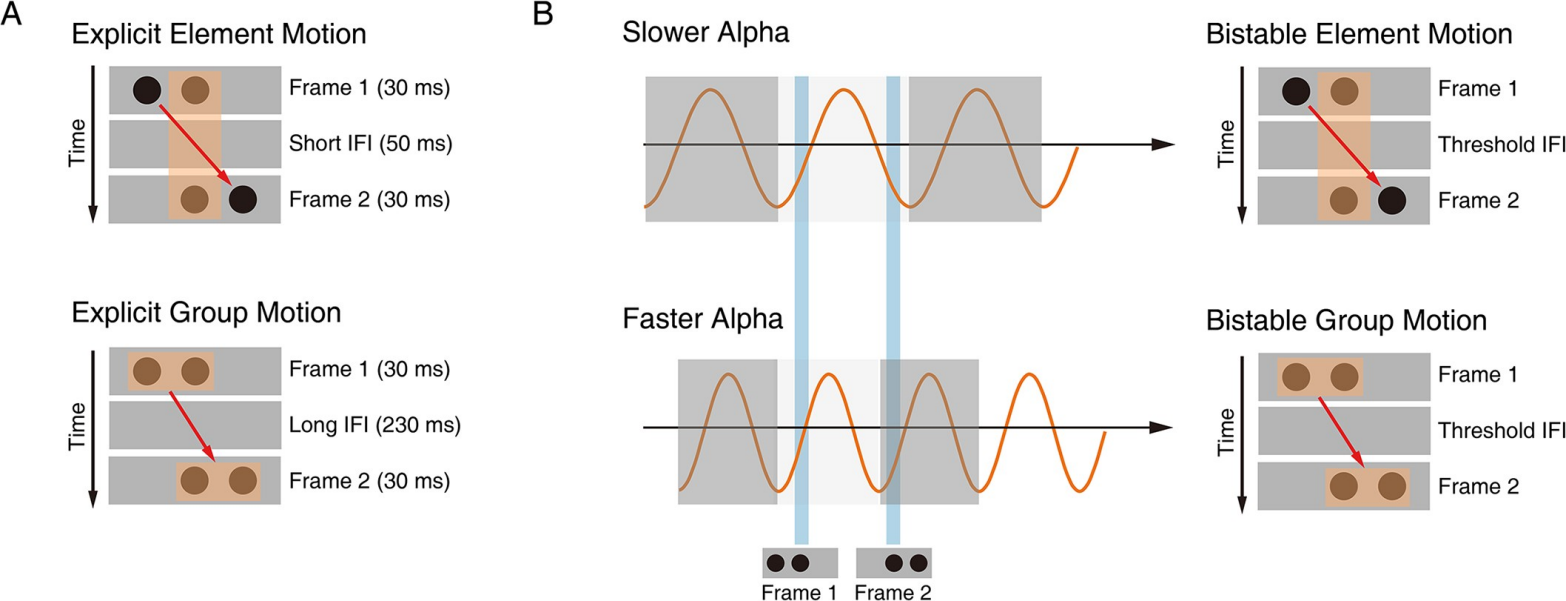
Schematic demonstration of the temporal and spatial grouping in the Ternus display and their hypothetical relations to the alpha cycles. (A) Upper panel: explicit EM at the short IFI (50 ms). At the short IFI, the shared central disk between the two frames is temporally grouped as the same object, resulting in explicit EM percepts. The central disk is perceived to remain still at the central location, and the two lateral disks are perceived to jump from one side to the other (see also S1 Video). Lower panel: explicit GM at the long IFI (230 ms). At the long IFI, the two disks within each of the two frames are spatially grouped, respectively, resulting in explicit GM percepts. The two disks in the first frame are perceived to move together as a group towards the second frame in a manner consistent with the physical displacement (see also S2 Video). (B) Upper panel: bistable EM percepts at the threshold IFI. The lower the PAF, the higher possibility the two consecutively presented frames will fall in the same alpha cycle, resulting in the EM percepts. Lower panel: bistable GM percepts at the threshold IFI. The higher the PAF, the two frames will more likely fall in different alpha cycles, resulting in the EM percepts. EM, element motion; GM, group motion; IFI, interframe interval; PAF, prestimulus alpha frequency.

**Fig 2:**
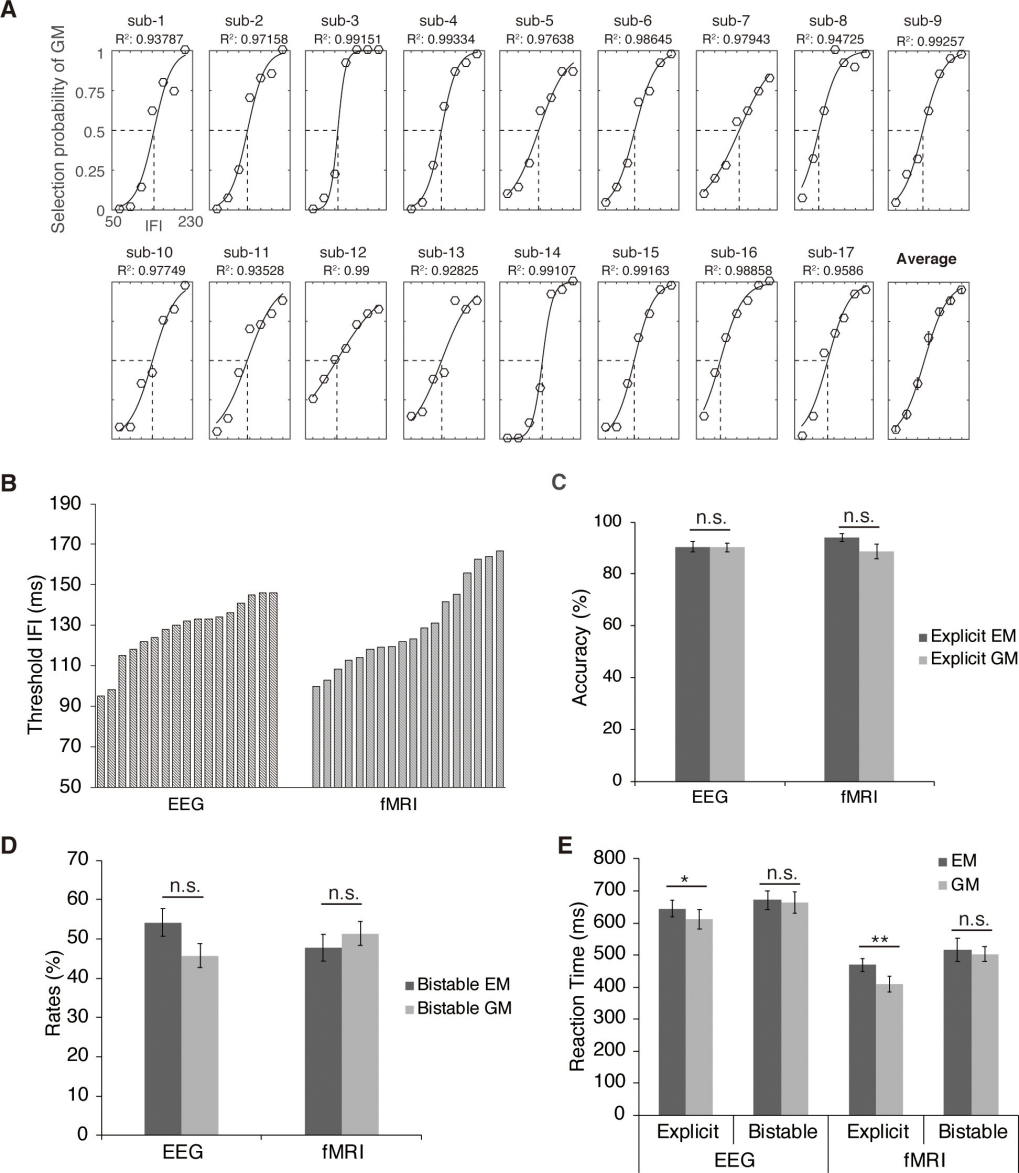
Behavioral results. (A) Psychometric curves fitted on the psychophysical data for each participant in the EEG experiment. The transition IFI threshold in each individual subject was defined as the IFI corresponding to the point of 50% reported GM/EM percepts on the fitted logistic function. All curves fitted well to the selection probability of GM (all R^2^ values > 0.9). (B) Threshold IFIs derived from the psychophysical procedure for each participant in the EEG and fMRI experiment, respectively. (C) Accuracy rates of the explicit EM and GM trials averaged across all the participants in the EEG and fMRI experiment, respectively. (D) Rates of the bistable EM and GM trials averaged across all the participants in the EEG and fMRI experiment, respectively. (E) RTs relative to the onset of frame 2 for all the experimental conditions in the EEG and fMRI experiment. The error bars indicate ±1 SEM. *p < 0.05, **p < 0.01. Underlying data available at https://osf.io/tze94/. EEG, electroencephalography; EM, element motion; fMRI, functional magnetic resonance imaging; GM, group motion; IFI, interframe interval; RT, reaction time.

The process of perceptual inference can be well illustrated by the phenomenon of bistable apparent motion in the Ternus display [20,21], in which subjective perception spontaneously alternates between spatial and temporal grouping interpretations of a constant ambiguous dynamic visual scene (Fig 1). The human brain adopts two major strategies of perceptual grouping to achieve perceptual coherence along the spatial and temporal dimension, despite the ever-changing visual inputs and the resulting fragmentary nature of the retinal image across space and time [22–26]. Spatially, grouping local visual elements into a holistic percept allows us to perceive scenes and objects as a whole rather than a meaningless collection of unconnected parts [27–29]. Temporally, successive discrete visual events unfolding in time could be grouped based on temporal proximity to perceive the stability of object identity and location [24,30,31]. In the classical Ternus display (Fig 1A), two horizontally spaced disks appear at shifted locations in two successive frames. Depending on the interframe interval (IFI), observers typically report two distinct percepts [32,33]. Temporal grouping is explicitly dominant at short IFIs: the central overlapping disks between the two frames are temporally integrated as one single disk, and the visual persistence of the central overlapping disk makes the lateral disk in frame 1 appear to jump across the central disk, i.e., element motion (EM) (Fig 1A, upper panel; S1 Video). On the other hand, spatial grouping is explicitly dominant at long IFIs: the two disks within each frame are spatially grouped and perceived as moving together as a group, i.e., group motion (GM) (Fig 1A, lower panel; S2 Video). Most critically, when the IFI reaches a certain psychophysical threshold, the Ternus display becomes ambiguous/bistable: the report of EM (temporal grouping) versus GM (spatial grouping) percepts randomly fluctuates on a trial-by-trial base, resulting in comparable proportions of GM and EM percepts (Fig 2A) [24,33]. Interestingly, the typical transition IFI threshold between temporal and spatial grouping in the Ternus display occurs around a time window of approximately 100 ms [33,34] (see Fig 2A and 2B), which corresponds to the average cycle of occipital alpha-band oscillations, with peak frequencies ranging between 8 and 13 Hz (i.e., 70- to 120-ms cycle).

The alpha oscillations, as one of the most predominant oscillations in the visual system, are considered as one underlying mechanism of perceptual cycles by gating the transient temporal windows of perception [35–39]. Accordingly, accumulating evidence shows that the phase of ongoing alpha oscillations reflects cyclic shifts of neuronal excitability [40–42] and predicts not only behavioral performance [15,43–45] but also a variety of subsequent neural signals related to stimulus processing [16,46,47]. Besides the phasic effects, the peak frequency of alpha-band oscillations predicts reaction times (RTs) [48] and variations in temporal resolution of perception [36,49–51]. The phasic and frequency effect of alpha oscillations lead to the long-standing hypothesis that the alpha cycle provides the discrete temporal window of perceptual grouping: whether two stimuli are integrated into a single percept or segregated into separate events depends on whether they fall in the same cycle of the alpha oscillation [52,53].

In terms of the Ternus paradigm, if the two frames fall in the same alpha cycle, they will be temporally integrated, resulting in the EM percepts; if the two frames fall in different alpha cycles, they will be segregated temporally, and spatial grouping will take place separately in the two frames, resulting in the GM percepts. Especially when the sensory inputs become ambiguous at the transition IFI threshold (Fig 1B), we hypothesize that the intrinsic prestimulus alpha frequency (PAF) plays a critical role in determining whether the two frames are grouped over time or not, which accordingly affects the codependent spatial grouping process. Specifically speaking, lower PAFs (i.e., longer prestimulus alpha cycles, Fig 1B, upper panel) allow the two consecutively presented frames to fall in the same alpha cycle, resulting in the EM percepts, while higher PAFs (i.e., shorter prestimulus alpha cycles, Fig 1B, lower panel) allow the two frames to fall in different alpha cycles, resulting in the GM percepts. We thus predict that the PAF can affect the outcome of bistable perceptual grouping in the Ternus paradigm, with higher PAFs preceding the bistable GM than EM percepts.

Furthermore, we hypothesize that perceptual inference employs the intrinsic PAFs to predict the perceptual outcome in the bistable Ternus display. Specifically, the brain generates predictions towards the GM percepts according to higher PAFs since the higher PAFs make it more possible for the two frames to fall in different alpha cycles. On the other hand, the brain generates predictions towards the EM percepts according to lower PAFs since the lower PAFs make it more possible for the two frames to fall within the same alpha cycle. Under the framework of perceptual inference, combining the specific prediction and forthcoming inputs, perceptual inference towards one specific percept will be made [54–56]. The perceptual inference is efficient if it is consistent with the subsequently perceived percept but inefficient if inconsistent. We thus predict that the efficiency of the perceptual inference may bias neural representations of the perceived percepts with the progress of time. In particular, the efficient perceptual inference may induce the corresponding representation pattern underlying the predicted percepts even before the actual presentation of the stimuli. Alternatively, if perception was based solely on sensory inputs, one would assume that neural representations underlying the integrated percepts are induced only after the actual presentation of the stimuli. To distinguish between the above hypotheses, we adopted electroencephalography (EEG) in healthy adults and intracranial recordings in epileptic patients and used multivariate decoding techniques on the EEG data to further probe the representational content of neural signals in a time-resolved manner.

## Results

### Behavioral performance

In the EEG (*n* = 17), intracranial (*n* = 4), and functional magnetic resonance imaging (fMRI) (*n* = 18) experiments, participants were asked to report the perceived EM versus GM percepts after viewing 1) the explicit EM stimuli with the short IFI, 2) the explicit GM stimuli with the long IFI, and 3) the bistable stimuli with the transition IFI threshold (Fig 1). The transition IFI threshold, at which equal proportions of EM and GM trials were reported, was determined individually for each participant prior to the main experiment (Fig 2A; see Materials and methods). The individual IFI threshold for each participant, estimated by a psychometric function fitted to the participant’s responses at each of the seven IFIs (see details in Materials and methods, Figs 2A and S1), was shown in Fig 2B for the EEG and fMRI experiments, respectively. A two-sample *t* test showed no significant difference between the two experiments in terms of the group mean IFI threshold, *t*_(33)_ < 1.

In the two explicit conditions, the mean accuracy rates in the explicit EM and explicit GM condition were comparable and both above 85% in both the EEG, *t*_(16)_ < 1, and the fMRI experiment, *t*_(17)_ < 1 (Fig 2C). The accuracy rate of the explicit trials was taken as an indicator of whether a participant could indeed clearly discriminate between the two different types of percept. Our data indicated that the participants could clearly distinguish the two explicitly different percepts at the short versus long IFIs. RTs, however, were significantly slower in the explicit EM than the explicit GM condition in both the fMRI experiment (*t*_(17)_ = 3.06, *p* < 0.01) and the EEG experiment (*t*_(16)_ = 3.73, *p* < 0.005) (Fig 2E). In the bistable condition, there was no significant difference between the bistable EM and the bistable GM conditions in terms of both the rates of choice (EEG experiment: *t*_(16)_ < 1, fMRI experiment: *t*_(17)_ < 1) (Fig 2D) and the RTs (EEG experiment: *t*_(16)_ < 1, fMRI experiment: *t*_(17)_ < 1) (Fig 2E). In addition, behavioral performance of the four epileptic patients with depth electrodes showed similar patterns as the healthy participants (see S1 Table).

### Neurophysiology results

#### PAF predicted the outcome of bistable perceptual grouping

**Fig 3:**
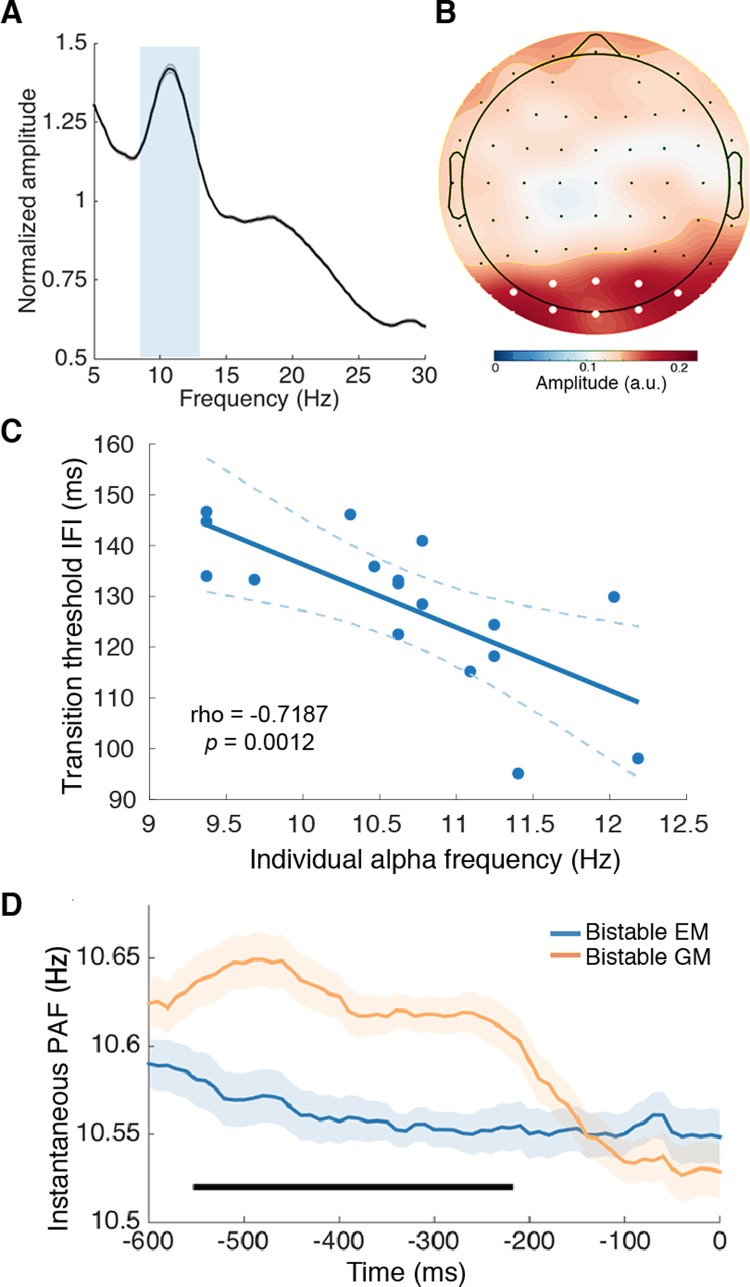
Relationship between the PAF and bistable perceptual grouping. (A) The normalized amplitude obtained through an FFT from all the bistable trials (from –800 to 0 ms relative to the onset of the first frame) collapsed over all electrodes in all the participants revealed a clear peak at the alpha frequency. The gray shading indicates ±1 SEM. The light gray rectangle indicates the selected frequency band. (B) The amplitude topographic map of the selected alpha frequency band (8–13 Hz) revealed a clear posterior scalp distribution. The selected posterior electrodes are indicated with white dots. (C) The frequency of occipital alpha in each individual subject, derived from the prestimulus alpha activity, was significantly correlated with the individual transition IFI threshold. Dashed lines indicate 95% confidence intervals around the linear fit line. (D) Within-subject analysis of the instantaneous PAF revealed higher alpha frequency preceding the bistable GM trials than the bistable EM trials. Significant time points are indicated by the horizontal black bar (cluster-based correction, *p* < 0.05). Shaded regions denote ±1 within-subjects SEM. Underlying data available at https://osf.io/tze94/. a.u., arbitrary unit; EM, element motion; FFT, Fast Fourier Transform; GM, group motion; IFI, interframe interval; PAF, prestimulus alpha frequency; SEM, standard error of the mean.

The EEG data showed a clear peak in the alpha-band amplitude (8–13 Hz) (Fig 3A) and a posterior scalp distribution of alpha amplitude during the prestimulus period (–800 to 0 ms relative to the presentation of the first frame) for all the participants (Fig 3B). These results guided further analysis by confining the frequency of interest to 8–13 Hz and the region of interest to the posterior electrodes (Oz, O1, O2, POz, PO1, PO2, PO3, PO4).

To understand the relationship between the alpha frequency and the perceived percepts of the bistable perceptual grouping, we first calculated the between-subject correlation between the individual transition IFI thresholds and the individual prestimulus peak alpha frequency. The individual peak alpha frequency was calculated based on the maximal prestimulus posterior alpha amplitude of each participant [57]. Subsequently, the Pearson correlation between the individual alpha frequency and the individual transition IFI threshold was calculated. The two measures were significantly correlated (*n* = 17, *r* = –0.7187, *p* = 0.0012) (Fig 3C). The significant between-subject correlation suggests that the faster the alpha oscillations (i.e., higher alpha frequencies and shorter alpha cycles) in an individual, the shorter the IFI required for the two frames to fall in different alpha cycles, and thus it takes shorter IFI for the later GM percepts at the longer IFIs to take dominance over the earlier EM percepts at the shorter IFIs. On the other hand, the slower the alpha oscillations (i.e., lower alpha frequencies and longer alpha cycles) in an individual, the longer the IFI required for the two frames to fall in different alpha cycles, and thus it takes longer IFIs for the transition from the earlier EM percepts to the later GM percepts. In addition, under the hypothesis that the alpha frequency gates the time window of temporal integration, one may expect that an increase in the alpha cycle length will increase the threshold IFI by the same amount. We accordingly tested whether the slope of the fitted line between the individual alpha cycle length and the individual transition IFI threshold (S2 Fig), which represents an increased transition threshold IFI per increment of the alpha cycle, was significantly different from the hypothetical slope of 1. The results showed that the slope of the linear regression does not significantly differ from 1, *t* < 1, indicating that an increase in the alpha cycle was neither significantly higher nor lower than an increase in the transition threshold of the IFI.

Moreover, if, as we predicted, slower alpha cycles lead to higher possibilities that the two frames will fall in the same alpha cycle (i.e., temporal grouping, Fig 1B, upper panel) while faster alpha cycles lead to higher possibilities that the two frames will fall in different alpha cycles (i.e., spatial grouping, Fig 1B, lower panel), trial-by-trial variance in the PAF within each subject should predict the outcome of the bistable perceptual grouping, with higher PAFs on the bistable GM (spatial grouping) than the bistable EM (temporal grouping) trials. To test the above prediction, we analyzed time-resolved changes in the prestimulus derivative of the phase angle time series (see Materials and methods), which corresponds to the instantaneous frequency of a signal within a band-limited range [58]. For each subject, we calculated and compared the instantaneous alpha frequency in the prestimulus window (–800 to 0 ms relative to the presentation of the first frame, and no poststimulus signal was included in the analysis) for the bistable GM and the bistable EM percept trials, respectively. Consistent with our predictions, the results showed that the PAF was significantly higher in the bistable GM than bistable EM trials, from about –550 to –210 ms relative to the presentation of the first frame, cluster-based correction (*p* < 0.05) (Fig 3D).

**Fig 4:**
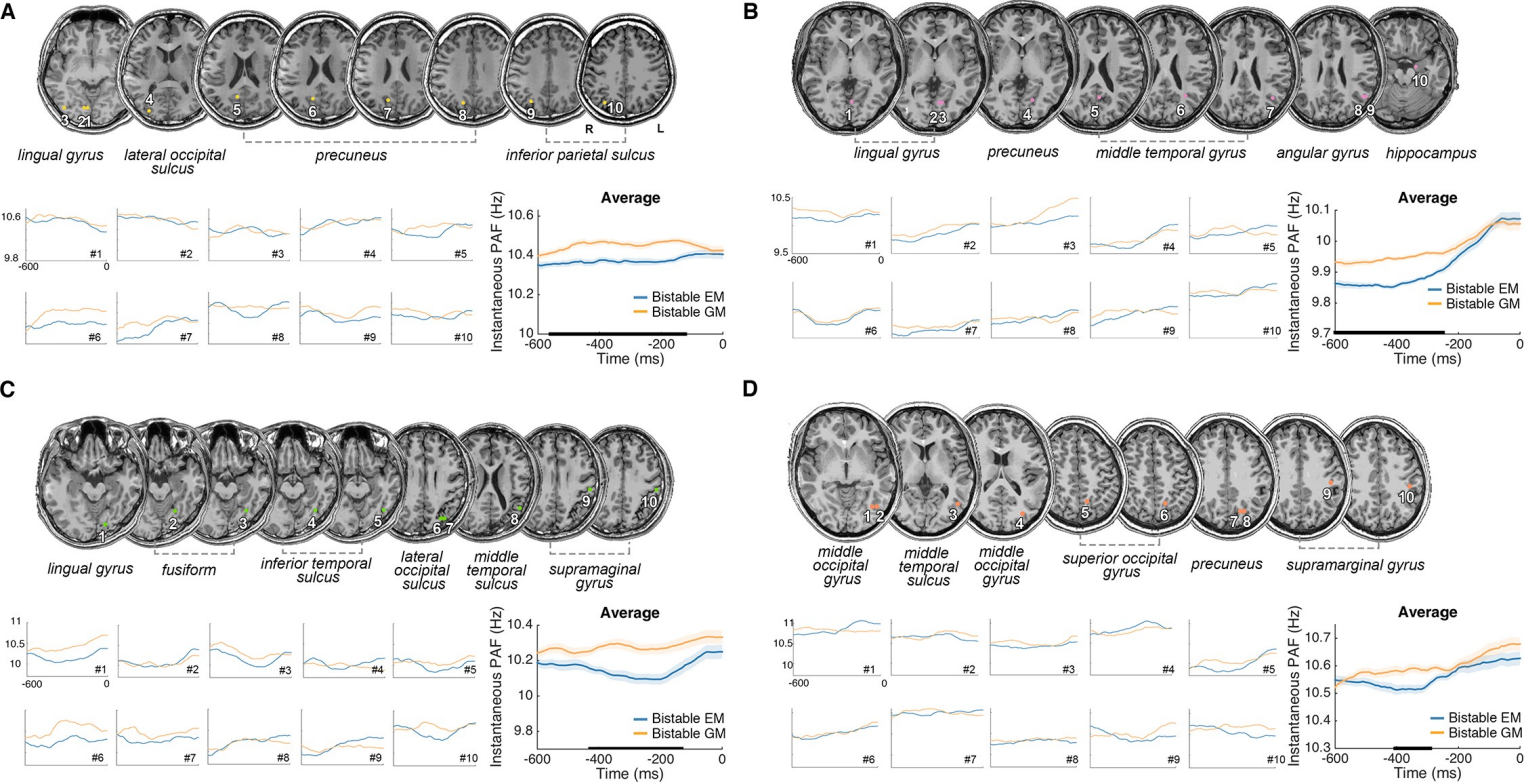
PAF effect in the four epileptic patients. Anatomical locations of the first ten contacts with the largest alpha (8–13 Hz) power from each of four patients (A, B, C, and D). Precise anatomical regions of the 10 selected contacts are marked on the coronal T1 slices of individual brains. Instantaneous PAF was calculated for the bistable EM and bistable GM trials, respectively, for each selected contact, and averaged across all the contacts. The alpha frequency preceding the bistable GM trials was higher than that preceding the bistable EM trials in most of selected contacts in each patient. In the averaged results, significant time points are indicated by horizontal black bars (cluster-based correction, *p* < 0.05). Shaded regions denote ±1 SEM. Underlying data available at https://osf.io/tze94/. EM, element motion; GM, group motion; PAF, prestimulus alpha frequency; SEM, standard error of the mean.

To further test the consistency of the above PAF effect and its precise anatomical origins, we collected intracranial data from four epileptic patients with depth electrodes. For each patient, according to the present frequency of interest at 8–13 Hz, we computed the alpha amplitude for each and every contact and then selected the first 10 contacts with the strongest alpha amplitude. The anatomical locations of these selected contacts were mostly located in the occipital and parietal regions (Fig 4), which was consistent with the posterior scalp distribution of alpha in the EEG experiment (Fig 3B).

We subsequently performed further analysis on the within-subject trial-by-trial variance of instantaneous PAF in the selected contacts. The instantaneous PAF of the bistable EM and GM trials was further computed for each of the 10 contacts with the strongest alpha power, using similar methods as those for the EEG data analysis. The results showed that most of the selected contacts exhibited the trend of higher PAF in the bistable GM than bistable EM trials (Fig 4). For all four patients, in the group mean of the 10 contacts with maximal alpha amplitude, the PAF was significantly higher for the bistable GM percepts than the bistable EM percepts using cluster-based permutation test (Fig 4A, patient 1: from about –500 to –110 ms relative to the presentation of frame 1; Fig 4B, patient 2: from about –470 to –180 ms; Fig 4C, patient 3: from about –600 to –250 ms; and Fig 4D, patient 4: from about –410 to –300 ms, cluster-based correction, *p* < 0.05).

#### PAF biased poststimulus neural representation by inducing preactivation of the subsequently reported bistable percepts

**Fig 5:**
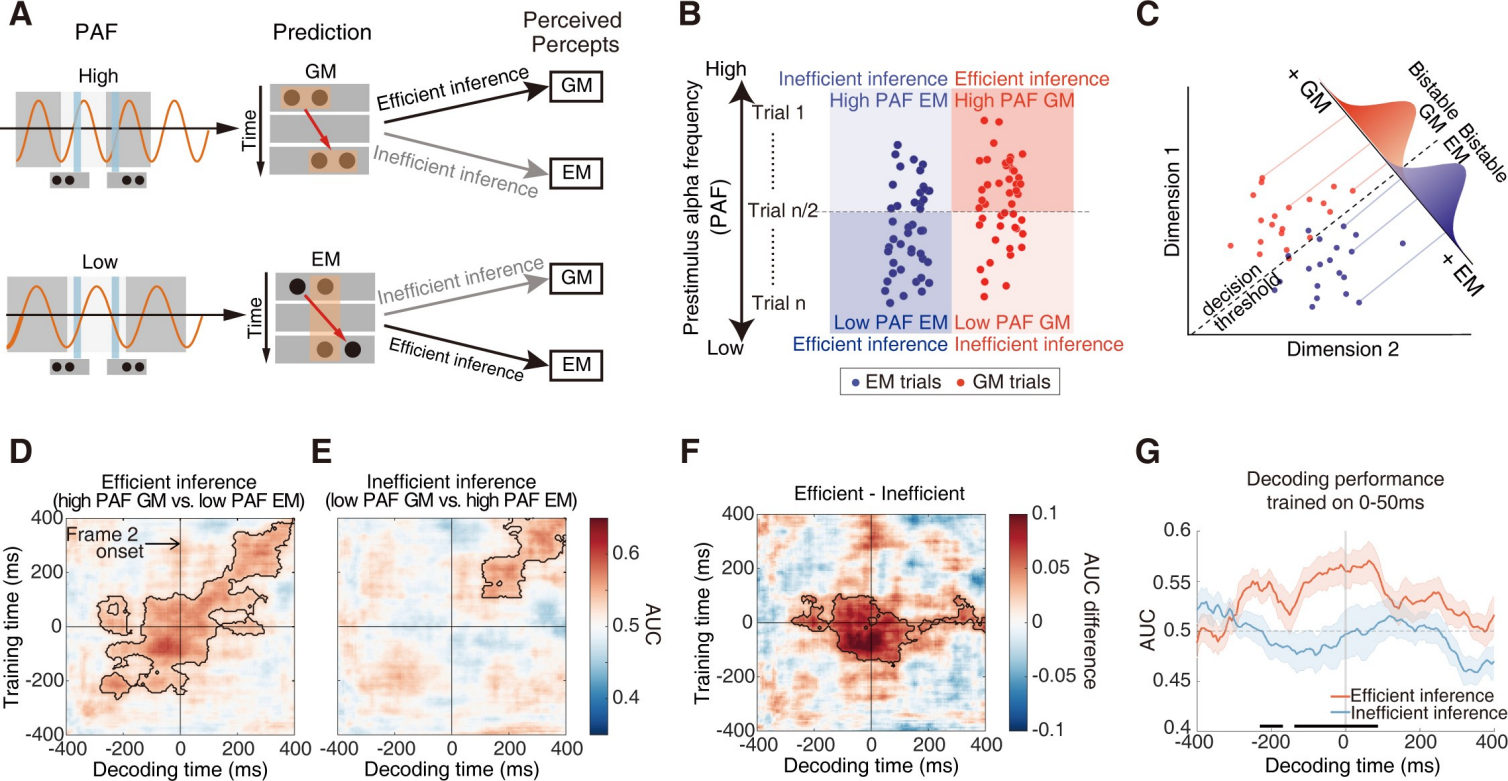
Hypothesis and results of decoding results of the EEG data. (A) Hypothesis on the efficient versus inefficient inference. Upper: predictions towards the GM percepts will be generated based on higher PAFs since it is more possible for the two frames to fall in different alpha cycles. Lower: predictions towards the EM percepts will be generated based on lower PAFs since it is more possible for the two frames to fall within the same alpha cycle. Conjointly considering the specific prediction and the forthcoming bottom-up inputs, perceptual inference towards one specific percept will be made. The perceptual inference is defined as efficient if it is consistent with the perceived percept and inefficient if inconsistent. (B) Post hoc trial categorization procedure for the decoding analysis. All the bistable trials were first sorted based on their PAF values. The bistable trials with a PAF higher than the PAF of the median trial (trial *n*/2, *n* = the total number of bistable trials) were considered as the high PAF trials, while the bistable trials with a PAF lower than the PAF of the trial *n*/2 were considered as the lower PAF trials. Therefore, conjointly based on the PAF (high versus low) and the perceptual outcome (EM versus GM), the bistable trials were categorized into the following four types: (1) the high PAF GM trials, (2) the low PAF EM trials, (3) the low PAF GM trials, and (4) the high PAF EM trials. The former two types of trials were assigned to the efficient inference condition since the prior perceptual inference based on the PAFs was consistent with the subsequently perceived percept, while the latter two types of trials were assigned to the inefficient inference condition since the perceptual inference was inconsistent with the perceptual outcome. (C) A hypothetical 2D activation space for the EEG signals representing the bistable EM and GM percepts. Activation patterns for each percept are projected onto a discriminant axis, which differentiates the two percepts. A decision boundary placed along the axis allows for classification between the bistable EM and GM percepts. The overlap of the Gaussian distributions reflects “decision noise.” More distant representations produce less noise and result in higher decoding accuracy. (D) Temporal generalization matrices for the efficient inference condition. (E) Temporal generalization matrices for the inefficient inference condition. (F) Temporal generalization matrices for the differential contrast between the efficient versus inefficient conditions. Columns in the images are the time points the classifier was trained, and rows are the time points the classifier was tested. Color values indicate decoding accuracy in terms of AUC (D, E) or AUC difference (F). The contour with the black line indicates the significant cluster, *p* < 0.05. (G) Decoding performance over time with training time 0–50 ms after the onset of frame 2. For the purpose of visualization, the figure shows a row in the temporal generalization matrix in (F) at the training time at which we see a significant cluster of generalization difference before the onset of frame 2. Significant generalization time points are indicated with horizontal black bars (*p* < 0.05). Time 0 indicates the onset of frame 2. Shaded regions indicate ±1 SEM. Underlying data available at https://osf.io/tze94/. AUC, area under the receiver operator characteristic; EEG, electroencephalography; EM, element motion; GM, group motion; PAF, prestimulus alpha frequency; SEM, standard error of the mean.

We subsequently investigated how the PAF biases the neural representations of the spatially versus temporally integrated percepts. The working hypothesis (Fig 5A) is that lower PAFs will result in efficient perceptual inference on the EM percepts and inefficient perceptual inference on the GM percepts. On the other hand, higher PAFs will result in efficient perceptual inference on the GM percepts and inefficient perceptual inference on the EM percepts. Accordingly, for the bistable GM trials with higher PAFs and the bistable EM trials with lower PAFs, we expected to observe biased neural representations of the predicted percepts in the neural signals not only after but also before the actual stimulus onset. To test the above hypothesis, we employed the temporal generalization method, a time-resolved decoding approach, to characterize how neural representations are dynamically transformed with the progress of time [59]. The classifiers used in the decoding analysis rely on boundaries through the high-dimensional activation space that maximally separate patterns of neural activity underlying different percepts (i.e., bistable EM versus bistable GM). Classifier performance should be better if the two representations in the activation space are clearly separated [60] (Fig 5C).

For the EEG data, all the bistable trials were first sorted according to the PAF and then half split into the high PAF and the low PAF sessions (see details in Materials and methods). Subsequently, the bistable EM and GM trials in the high PAF session were selected as the high PAF EM and GM trials, respectively (Fig 5B). Similarly, the bistable EM and GM trials in the low PAF session were selected as the low PAF EM and GM trials, respectively (Fig 5B). To exclude the potential confounds caused by the different number of trials, the trial number in each of the above four types of trials was matched (see details in Materials and methods). According to our hypothesis, the high PAF GM trials and the low PAF EM trials were designated as the efficient inference condition, while the low PAF GM trials and the high PAF EM trials were designated as the inefficient inference condition (Fig 5A). For each condition, we calculated the temporal generalization matrix, which contained the decoding performance between the bistable EM and bistable GM trials over time (quantified by the area under the receiver operator characteristic, i.e., AUC, using the leave-one-out cross-validation method). If the process of perceptual integration simply depends on sensory inputs, perceptual grouping between the two frames should happen only after the actual presentation of the second frame. We thus time locked the temporal generalization matrix to the presentation of the second frame to investigate how neural representations of the spatially versus temporally integrated percepts were encoded with the progress of time relative to the presentation of frame 2.

For the efficient inference condition, neural representations of the bistable EM versus GM percepts could be successfully discriminated both before and after the onset of the second frame (Fig 5D, from –280 ms to 400 ms relative to the second frame, cluster-based correction, *p* < 0.05). For the inefficient inference condition, however, the two types of percept could be successfully discriminated only after the onset of the second frame (Fig 5E, from 100 ms to 400 ms after the second frame, cluster-based correction, *p* < 0.05). By directly comparing the decoding performance between the efficient versus inefficient inference condition, we found significantly better decoding performance in the efficient than inefficient inference condition, not only after but also before the onset of the second frame (a significant cluster from training time –150 to 100 ms and decoding time –280 to 160 ms, cluster-based correction, *p* < 0.05) (Fig 5F). In particular, the neural signals 0–100 ms after the onset of frame 2 could be significantly better generalized to the neural signals 0–280 ms before the onset of frame 2 in the efficient than inefficient inference condition and vice versa (0–140 ms before frame 2 onset being generalized to 0–160 ms after frame 2). For demonstration purposes, these generalized signals were further illustrated, for example, when the decoder was trained 0–50 ms after frame 2 (Fig 5G): in the efficient inference condition, neural signals from 0 to 220 ms before frame 2 were similar to those evoked by the actual onset of frame 2. Please note here, the 0–50 ms poststimulus training time window in Fig 5G was just one representative time window taken from Fig 5F and was not the only significant time window. Instead, the significant effects extend from 0 to 100 ms after the actual presentation of frame 2 (Fig 5F). Taken together, the above decoding results suggested that the efficient perceptual inference, based on the intrinsic PAFs, improved the readout of poststimulus temporally versus spatially integrated representations by preactivating the percept-like signals even before the actual onset of frame 2.

#### Reduced prestimulus alpha power for the bistable EM percept

**Fig 6:**
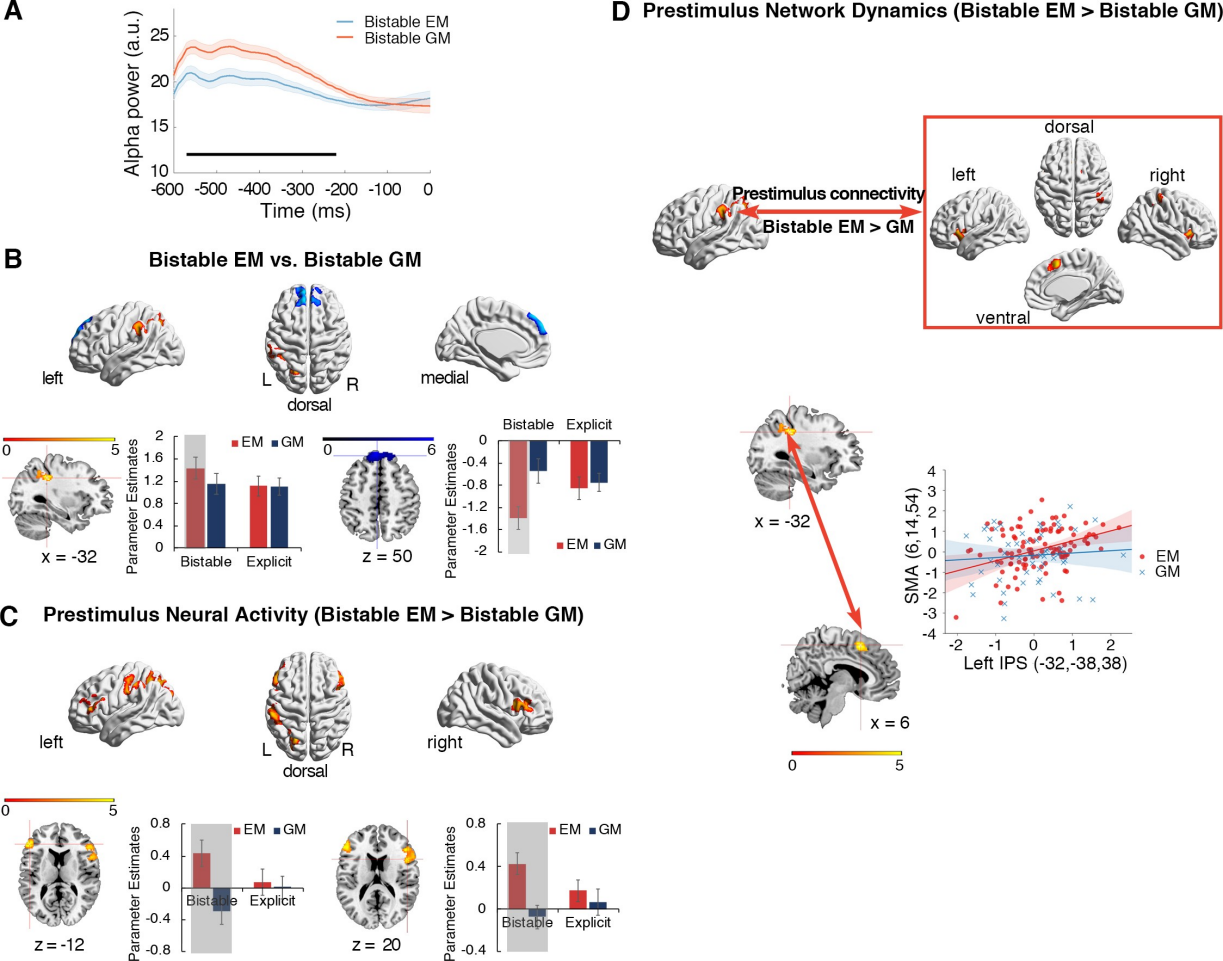
Prestimulus alpha power in the EEG data and pre- and peristimulus neural activity and network dynamics in the bistable trials of the fMRI data. (A) Occipital prestimulus alpha power was significantly lower in the bistable EM trials than in the bistable GM trials. Significant time points in the prestimulus period are indicated with the black bar (cluster-based correction, *p* < 0.05). Shaded regions denote ±1 within-subject SEM. (B) Red: left IPS showed significantly higher neural activity in the bistable EM than bistable GM trials. Blue: MPFC in DMN was significantly more deactivated in the bistable EM than bistable GM trials. Parameter estimates in the four experimental conditions were extracted from the activated left IPS (lower panel, left) and the MPFC cluster (lower panel, right). The shaded condition is the bistable EM condition that drives the significant neural contrasts. The error bars are ±1 SEM. (C) Increased prestimulus neural activity of the bistable EM trials, compared to the prestimulus neural activity of the bistable GM trials. Left IPS and bilateral IFG were significantly activated. Parameter estimates, indicating the height of neural activity in the previous trials (Trials N-1) of the bistable and the explicit trials, were extracted from the bilateral IFG. The height of neural activity in the trials prior to the bistable EM trials was higher than in the trials prior to the bistable GM trials (the two shaded conditions). (D) PPI analysis based on prestimulus neural activity in the left IPS, with the contrast “bistable EM > bistable GM” as the psychological factor. The left IPS showed enhanced neural coupling with sensorimotor and frontal areas during the prestimulus period of the bistable EM trials, compared to the prestimulus period of the bistable GM trials. For example, in a representative participant, mean corrected prestimulus neural activity in the SMA is shown as a function of mean corrected prestimulus activity in the left IPS (i.e., the first principal component from a sphere of 4 mm radius) for the bistable EM trials (red dots and lines) and bistable GM trials (blue dots and lines), respectively. Underlying data available at https://osf.io/tze94/. a.u., arbitrary unit; DMN, default-mode network; EEG, electroencephalography; EM, element motion; fMRI, functional magnetic resonance imaging; GM, group motion; IFG, inferior frontal gyrus; IPS, intraparietal sulcus; MPFC, medial prefrontal cortex; PPI, psychophysiological interaction; SEM, standard error of the mean; SMA, supplementary motor area.

In addition to the alpha frequency effect, we further tested whether the prestimulus alpha power could potentially influence the bistable perception. We calculated the prestimulus occipital alpha power, relative to the onset of the first frame, for the bistable EM and bistable GM trials, respectively (see also Materials and methods). Significantly lower prestimulus alpha power was found in the bistable EM than bistable GM trials, from –580 to –220 ms relative to the onset of frame 1 (Fig 6A).

#### fMRI data: Enhanced prestimulus activity and network dynamics in the frontoparietal network for the bistable EM percept

For the fMRI data, we first identified the specific neural substrates underlying the bistable spatially versus temporally integrated percepts by directly contrasting the bistable EM and GM trials. Compared to the bistable GM trials, the bistable EM trials induced stronger positive activations in the left intraparietal sulcus (IPS) (Fig 6B, upper panel, red; S2 Table), and stronger deactivations in the medial prefrontal cortex (MPFC) of the default-mode network (DMN) (Fig 6B, upper panel, blue; S2 Table). As shown in the mean parameter estimates extracted from the activated clusters (Fig 6B, lower panel), neural activity increased in the left IPS and decreased in the MPFC, specifically in the bistable EM trials.

To further localize the specific neural regions in which prestimulus neural activity predicts the outcome of bistable perceptual grouping, we compared neural activity in the trials prior to the bistable EM and GM trials (see Materials and methods). Left inferior parietal cortex and bilateral inferior frontal gyrus (IFG) showed significantly enhanced prestimulus neural activity of the bistable EM trials, compared to the bistable GM trials (Fig 6C, upper panel). For example, the extracted mean parameter estimates in bilateral IFG showed that prestimulus neural activity was higher for the bistable EM than the bistable GM trials but was comparable between the explicit EM and the explicit GM trials (Fig 6C, lower panel; S2 Table). The left inferior parietal cortex showed similar patterns. No significant activations were revealed in the reverse contrast, i.e., bistable GM > bistable EM. These results thus suggested that enhanced neural activity in the frontoparietal network prior to the presentation of the bistable stimuli predicted the bistable EM percepts.

In addition to the height of prestimulus neural activity, prestimulus network dynamics in the frontoparietal attention network may play a critical role in predicting the outcome of bistable perceptual grouping as well. To address this, we compared the patterns of functional connectivity among the frontoparietal regions prior to the presentation of the bistable EM versus GM trials. Since the left IPS exhibited specific selectivity towards the bistable EM percepts during both the pre- (Fig 6C) and the poststimulus (Fig 6B) period, we used the left IPS as the seed region to perform the network analysis, focusing on the prestimulus period.

Psychophysiological interaction (PPI) analysis treated prestimulus activity in the left IPS as the physiological factor and the contrast between the two bistable percepts (bistable EM versus bistable GM) as the psychological factor. In this way, we aimed to calculate how prestimulus changes in the functional connectivity of the left IPS predict the subsequent bistable EM versus GM percepts. The results showed that prestimulus functional connectivity between the left IPS and the frontal regions was significantly enhanced for the bistable EM trials compared to the bistable GM trials (Fig 6D and S3 Table). No significant activations were found in the reverse contrast, i.e., bistable GM > bistable EM. Therefore, the enhanced dynamics in the frontoparietal network prior to the presentation of the bistable stimuli predicted the subsequent subjectively perceived EM percepts.

## Discussion

By using EEG, intracranial recordings, and fMRI, we investigated how the frequency of alpha-band oscillations acts as the critical neural dynamics that accommodate the temporal and spatial grouping during ambiguous perception in the Ternus paradigm and, more importantly, how the brain makes predictions, based on intrinsic alpha frequency, to resolve perceptual ambiguity. At the behavioral level, comparable task performance/judgment difficulty was revealed between the bistable temporal and spatial grouping condition (Fig 2D and 2E). Therefore, any neuronal difference between the two bistable conditions cannot be attributed to differences in judgment difficulty. At the neural level, both within and between subjects, peak prestimulus frequency of alpha oscillations in the occipitoparietal regions predicted the bistable temporal versus spatial grouping (Figs 3 and 4). Moreover, efficient perceptual inference, based on spontaneous variance in the intrinsic PAFs, induced a representation of the subsequently reported bistable percept in the neural signals before the actual appearance of the second frame, indicating a preactivation of the subjectively perceived bistable percepts (Fig 5). Based on the above observations, we propose that the alpha frequency gates the time window for perceptual grouping and perceptual inference based on intrinsic alpha frequency biased poststimulus neural representations by inducing preactivation of the predicted percepts. In addition, the reduced prestimulus alpha power (Fig 6A), together with enhanced prestimulus blood-oxygen-level–dependent (BOLD) activity and network dynamics in the frontoparietal network (Fig 6C and 6D), predicted bistable EM rather than GM percepts.

It has been proposed that perception is discrete and cyclic in a manner of perceptual cycles [15,16,45,61,62]. Accumulating recent evidence showed that perceptual performance depends on the frequency of the critical rhythm at around the onset time of stimuli [50,51,63]. A higher frequency of the brain oscillations should be equivalent to a faster frame rate of discrete perception and vice versa. Accordingly, lower alpha frequency was reported to be associated with poorer temporal resolution [50,51], as if the slower frame rate of perception made two successive flashes more likely to fall within the same frame and thus be perceived as one [61]. In the present Ternus paradigm, the intrinsic alpha frequency determines whether the two frames are integrated over time (i.e., EM) or not (i.e., GM). Specifically, when the alpha frequency is relatively slow (i.e., longer alpha cycles) to cover both spatially and temporally segregated information segments, temporal grouping between the frames dominates over spatial grouping, resulting in the EM percepts (Fig 1B, upper panel; Fig 3C and 3D). On the other hand, when the alpha frequency is relatively high (i.e., shorter alpha cycles) to cover only spatially segregated information segments, spatial grouping with the frames dominates, resulting in the GM percepts (Fig 1B, lower panel; Fig 3C and 3D).

The intracranial data further confirmed the above alpha frequency effect in distributed visual areas including both the dorsal and ventral visual stream, such as the primary and secondary visual areas in the lingual gyrus, higher-order areas in the fusiform gyrus, the lateral occipital cortex (LOC), the middle temporal gyrus (MT), and the IPS (Fig 4). The dorsal occipitoparietal areas, such as the inferior IPS, have been associated with perceptual integration of multiple elements and object representations [64,65]. The ventral visual areas, such as LOC, have been found to be involved in object recognition [66]. Moreover, it has been well documented that the MT area is highly responsive to visual motion and codes highly specialized representations of visual information [67–69], which is putative for generating apparent motion percepts [70,71]. The present results further suggest that the alpha frequency effect is a ubiquitous property of the visual system, which is involved in representing coherent object motion percepts. It has been revealed that neural oscillations could create temporal windows that favor the communication between neurons [72,73]. The common alpha frequency effect in distributed visual systems may drive the communication between neuronal groups in these areas to effectively encode and organize the dynamic visual inputs and induce coherent apparent motion percepts.

Please note, for both the present EEG and the intracranial results, a very small within-subject variance in the PAF (about 0.1 Hz in the EEG data, Fig 3D, and about 0.2 Hz in the intracranial data, Fig 4) was associated with qualitatively different perceptual percepts. Based on our hypothesis, at the within-subject level, the most critical factor that causes different perceptual outcomes is the perceptual inference built through the intrinsic alpha frequency, but not the absolute alpha frequency per se. It has been suggested before that the perceptual inference is very sensitive to subtle changes in the intrinsic brain states [74,75]. Therefore, in the present study, a slightly lower alpha frequency could be enough to induce a perceptual inference towards the EM percept, while a slightly higher alpha frequency could be enough to induce an inference towards the GM percept. In addition, the small within-subject frequency effect is consistent with previous studies showing small frequency modulations [51,58]. Technically speaking, this small effect might result from the fact that the alpha frequency data derived from the EEG and intracranial signals reflect the summed activity of both the task-relevant and the task-irrelevant neuronal populations. Therefore, the observed effect could be attenuated by the noises from the task-irrelevant neuronal populations [51].

The generative models of perceptions commonly consider the brain as an unconscious inference machine that uses hidden states to predict observed sensory inputs [55,76]. Although there have been some detailed theories on the neural basis of the underlying computations of this system [77], it still lacks direct empirical evidence about how the brain uses its intrinsic states to build up specific prediction signals for perceptual inference. In the present study, even with the sensory inputs (the two frames with a threshold IFI) being kept constant in the bistable condition, the subjective perception varies between the EM and GM percepts on a trial-by-trial base, thus suggesting fluctuations in prior predictions. Please note, the definition of prediction in the present study stands for “internal model’s prediction” under the general perceptual inference framework [54,74], which is different from the term of “top-down prediction” manipulated in the field of cognitive neuroscience and psychology [78,79]. The former one represents the priors in the Bayesian framework and includes any factors that can provide prior information [6,55]: the perceptual prediction based on intrinsic PAFs in the present study is an example of this type of prediction. On the other hand, the latter term of “top-down prediction” is associated with the top-down control mechanisms in the higher-order brain areas, which is not directly supported by the data in the present study.

The present Ternus display puts the brain under the explicit contextual information that there are two possible apparent motion percepts, i.e., GM versus EM. Moreover, the alpha peak frequency, which provides the critical window for perceptual integration, is widely considered as one putative marker of an individual’s intrinsic state [80,81]. Therefore, perception is able to employ the current intrinsic alpha frequency to build prior probability of predictions about the most possibly perceived apparent motion percepts (Fig 5A, left panel). The perceptual inference is efficient if it is consistent with the perceptual outcome, i.e., in the high PAF GM trials and the low PAF EM trials; the inference is inefficient if it is inconsistent with the perceptual outcome, i.e., in the high PAF EM trials and the low PAF GM trials (Fig 5A, right panel). Our results showed that the peak alpha frequency not only predicted the outcome of bistable perceptual grouping (Figs 3C, 3D and 4) but also modulated the fidelity of neural representations of the integrated percepts (Fig 5). Compared to the inefficient inference, neural representations of the bistable EM versus GM percepts could be more robustly decoded under the efficient inference (Fig 5D, 5E and 5F), suggesting that the efficient inference based on intrinsic PAFs enhanced the fidelity of neural representations of the predicted percepts. More interestingly, under the efficient inference, the neural signals evoked by the actual presentation of the second frame could be readily read out from the neural signals even before the presentation of the second frame (Fig 5F and 5G), suggesting a preactivation of the predicted percepts. These results thus fundamentally advance our mechanistic understanding on how the alpha frequency builds up specific prediction signals for perceptual inference: perceptual predictions on the spatially versus temporally integrated percepts are generated based on variation in the intrinsic PAFs, which induces preactivated neural representations that resemble the neural representations evoked by the actual stimuli. Please note, since time 0 in the decoding analysis was relative to the presentation of frame 2, the significant 0–100 ms poststimulus time window of the decoding analysis (Fig 5F) corresponds to about 100 (threshold IFI)–200 ms (threshold IFI + 100 ms) relative to the actual presentation of frame 1. It is thus possible that, about 150–200 ms after the presentation of frame 1, the participants have already generated conscious perceptual experiences of the predicted percepts under the modulation of perceptual prediction [14,82]. However, since the whole significant poststimulus time window still involves relatively early processing phases around 100–150 ms after the presentation of frame 1, one alternative interpretation is that the present relatively early poststimulus time window might reflect early neural mechanisms such as the iconic memory [83].

Since the explicit EM percepts are observed at the short IFI while the explicit GM percepts are observed at the long IFI (Figs 1A and 2A), and since the bistable EM percepts involve higher alpha frequency than the bistable GM percepts (Fig 3D), it is possible that shorter time frames and accordingly faster temporal processing are involved in the EM percepts, which may demand more efficient communication of information through the brain. It has been correspondingly suggested that attention facilitates fast temporal processing [84–86]. Therefore, one hypothesis is that the bistable EM percepts may require more frontoparietal attentional network involvement than the bistable GM percepts. Alternatively, in contrast to EM, which is more a temporal matching, GM is more a gestalt/global matching of objects, which ignores retinotopic correspondence in favor of object-based grouping [32,87,88]. Such high-level, nonretinotopic, gestalt grouping of GM might be expected to require more frontoparietal involvement as opposed to the occipital regions, which might be sufficient for short-lived, retinotopically organized grouping [89]. Our fMRI results provide supporting evidence to the former hypothesis: both increased prestimulus neural activity (Fig 6C) and increased prestimulus network dynamics (Fig 6D) in the frontoparietal network predicted the subsequent bistable EM (temporal grouping), rather than GM (spatial grouping), percepts. Moreover, the enhanced frontoparietal activations and DMN deactivations during the bistable EM trials (Fig 6B) indicated that bistable temporal grouping was more attention-demanding than bistable spatial grouping [90,91]. Consistent with the fMRI results, the prestimulus alpha power results (Fig 6A) also supported this conclusion by showing a lower prestimulus alpha power in the bistable EM than GM trials. Since it has been well documented that alpha power is an effective indicator of the level of attention engaged in a certain cognitive task (the higher the alpha power, the lower the level of attention) [81,92–94], the lower prestimulus alpha power during the bistable EM trials indicated higher level of attention. Therefore, the fMRI results, together with the prestimulus alpha power results, suggested that temporal grouping is more attention-demanding than spatial grouping in the Ternus paradigm.

To further understand the more general role of alpha frequency in perceptual grouping across both space and time rather than just see the specific effect with a single variant of the Ternus paradigm, future experiments with paradigms examining perceptual grouping at different levels of complexity and with regard to different visual attributes are still needed. In terms of the Ternus paradigm per se, the present study only focused on the temporal window of perceptual integration and the effect of perceptual inference, while there are other possible interpretations of this specific illusion, such as alternations between object versus group processing [32,33], between the use of top-down predictions (for example, trial history) [95], and between the use of different reference frames [96–98]. Moreover, other frequency-band oscillatory activities, such as the theta-band oscillations that have been suggested to be implicated in the perception of apparent motion [63] and temporal integration [99], might be involved in the present phenomenon as well, but the current research methods may not be sufficient enough to detect these significant theta effects. For example, in the intracranial experiment, since most of the implanted electrodes of the four patients were in the posterior brain regions with few electrodes in the higher-order areas, it remains unknown whether there is a significant theta effect in the higher brain areas, such as the frontal cortex [100,101].

To summarize, by adopting a Ternus display in which subjective perception fluctuates between temporally versus spatially integrated percepts, we showed that the occipitoparietal alpha frequency defines a temporal window for perceptual integration. Moreover, in the situation of efficient perceptual inference, neural representations of the predicted percepts based on the alpha frequency were preactivated before the actual presentation of the critical stimuli. Therefore, perceptual inference employs PAF-induced predictions to resolve perceptual ambiguity.

## Materials and methods

### Ethics statement

All the participants gave their informed consent prior to the experiment in accordance with the Declaration of Helsinki. The fMRI, the EEG, and the patient experiments were all approved by the Ethics Committee of School of Psychology, South China Normal University (06202015_TernusCQ). The placement of the depth electrodes was based solely on the clinical needs for the treatment of the patients and was thus independent of the purpose of the present study. This study did not add any invasive procedure to the intracranial recordings. All the participants were at least 18 years old and gave their written informed consent prior to the experiments.

### Participants

Nineteen adult participants (12 females, mean age of 19.6 years old) took part in the EEG experiment. Another group of 20 adult participants (12 females, mean age of 23.4 years old) took part in the fMRI experiment. Two participants in the EEG experiment were discarded because of excessive eye movement artifacts. One participant in the fMRI experiment was discarded because of low accuracy (less than 70%) in the explicit conditions, and another participant was discarded because of the excessive head movements during the scanning. Therefore, 17 participants in the EEG experiment and 18 participants in the fMRI experiment were included for further analysis. Additionally, four adult patients (two males, mean age of 24 years old) undergoing intracranial recordings with stereotactically implanted multilead electrodes (Guangdong Sanjiu Brain Hospital, China) for epilepsy treatment participated in the present study. Although the anatomical locations of the electrodes were different in each patient, we included the patients whose electrodes were implanted in the occipital and parietal regions. Patients who had destructive lesions such as tumor or encephalomalacia were excluded. All the participants were right-handed, with normal or corrected-to-normal visual acuity.

### Stimuli

Visual stimuli consisted of two consecutively presented frames of stimuli (frame 1 and frame 2), and each frame was presented for 30 ms (Fig 1). There was a blank period between the two frames, i.e., the IFI. The IFI could be either explicitly short at 50 ms or explicitly long at 230 ms or at the transition threshold, which was specific for each subject based on pre-experiment psychophysics. Each frame contained two horizontally arranged black disks (1.6° of visual angle in diameter) on a gray background. The center-to-center spatial distance between the two disks was 3° of visual angle. The two frames shared one common disk location at the center of the display. The location of the lateral disk of the first frame, either on the left or the right side of the shared central disk, was always opposite to the lateral disk of the second frame (Fig 1). Specifically speaking, frame 1 with left and central disks and frame 2 with right and central disks induced rightward apparent motion; frame 1 with right and central disks and frame 2 with left and central disks induced leftward apparent motion. The same set of stimulus parameters was adopted for the fMRI, the EEG, and the patient experiments. Depending on the IFI and participants’ online judgments in the bistable trials, there were four types of experimental trials: 1) the explicit EM trials (“Explicit EM”) with the short IFI of 50 ms; 2) the explicit GM trials (“Explicit GM”), with the long IFI of 230 ms; 3) the bistable trials with the threshold IFI, which were judged by the participants as the EM trials (“Bistable EM”); and 4) the bistable trials with the threshold IFI, which were judged by the participants as the GM trials (“Bistable GM”).

### Psychophysical procedures

To specify the 50% threshold of IFI for the bistable condition for each individual subject, we asked each participant to perform a psychophysical pretest before the main experiment. Prior to the psychophysics test, participants were shown demos of the explicit EM and GM conditions and performed a practice block with only explicit EM and GM trials until the accuracy reached no less than 95%. During the formal psychophysics test, the first frame was presented for 30 ms. After a variable IFI (seven levels: 50, 80, 110, 140, 170, 200, or 230 ms), the second frame was presented for 30 ms as well. Participants were asked to perform a two-alternative forced choice (2AFC) task in which they had to choose between the EM and the GM percept. For each IFI condition, the percentage of GM reports (i.e., “1 –percentage of EM reports”) was collapsed over the leftward and rightward motion directions. The seven data points (one for each IFI) were fitted into a psychometric curve using a logistic function [102]. The transition IFI threshold, i.e., the point at which EM and GM were reported with equal possibility, was calculated by estimating the 50% performance point on the fitted logistic function for each participant [102]. The individual transition threshold derived from the psychophysics test was then used as the IFI in the bistable trials of the subsequent main experiment. Differently from the EEG and fMRI experiment, in the intracranial experiment, an adaptive staircase procedure [103] was adopted to find the individual IFI threshold at which 50% of the stimuli were perceived as GM.

### Main experiment procedures

Participants were instructed to fixate at a central fixation throughout the experiment without moving their eyes. The experimental task was to discriminate the two types of motion by pressing two prespecified buttons on the response pad using the thumb of each hand, respectively. The mapping between the two response buttons and the two types of apparent motion percept was counterbalanced between participants.

In each trial, the first frame was presented for 30 ms, and after a variable IFI (50 ms, 230 ms, or the individual IFI threshold), the second frame was presented for another 30 ms. The fMRI experiment consisted of 440 trials in total, including 80 explicit EM trials, 80 explicit GM trials, 160 bistable trials, and 120 null trials. The null trials, in which only the central fixation cross was presented, were used as the implicit baseline. The participants were asked to rest for a short period of time (11 s, i.e., five repetition times [TRs]) after every 6 minutes’ task performance, which made three short periods of rest in total. During the three short rest periods, the scanner kept running, and a visual instruction “rest” was presented on the center of the screen throughout. One TR after the disappearance of the “rest” instruction, the behavioral task resumed. The EEG experiment consisted of four blocks, and each block included 40 explicit EM trials, 40 explicit GM trials, and 80 bistable trials, which were intermixed randomly, resulting in 640 experimental trials in total. A rest break was allowed between blocks. For the fMRI and EEG experiment, each trial was followed by a time interval that was selected randomly among 2,000, 2,250, 2,500, 2,750, and 3,000 ms. In the intracranial experiment, there were four blocks of 80 trials (320 trials in total), 10% of which were explicit EM and GM trials. The intertrial interval varied randomly between 1.5 and 2.5 s. In all the three experiments, the temporal order of all the trials was randomized for each participant individually to avoid potential problems of unbalanced transition probabilities. All participants completed a training section of 5 min before the recording.

### Recording and preprocessing of the EEG data

EEGs were continuously recorded from 64 Ag/AgCl electrodes (10–20 System) with BrainAmp DC amplifiers (low-pass = 100 Hz, high-pass = 0.01 Hz, and sampling frequency = 500 Hz). The vertical electro-oculogram was recorded by one electrode under the participants’ left eyes. All the electrode impedances were kept below 5 kΩ. Signals were referenced online to the unilateral mastoid. Offline processing and analysis were performed using EEGLAB [104] and customized scripts in MATLAB (The MathWorks, Natick, MA, USA). Data were down-sampled to 160 Hz, rereferenced to the average reference, epoched from –800 ms before the first frame to 1,000 ms after the first frame for the subsequent alpha frequency analysis, and re-epoched from –500 ms to 500 ms relative to the presentation of the second frame for the decoding analysis. Trials containing visually identified eye movements or muscle artifacts were excluded manually. Visually identified noisy electrodes were spherically interpolated.

### Acquisition and preprocessing of the intracranial data

Ten to 13 semirigid, multilead electrodes were stereotactically implanted in the four participants, respectively. All the electrodes have a diameter of 0.8 mm and contain 10–16 2-mm–wide and 1.5-mm–apart contacts. The precise anatomical location of each contact was identified by coregistering each participant’s postimplantation CT with the preimplantation 3D T1 image, using rigid affine transformations derived from FSL’s FLIRT algorithm [105]. Intracranial recordings were conducted using commercial video–intracranial monitoring system. The data were bandpass filtered online from 0.1 to 300 Hz and sampled at 1,000 Hz, using a reference contact located in the white matter. For the offline analysis, recording signals were down-sampled to 500 Hz. Contacts in the epileptogenic zones were excluded from further analyses. Each contact was rereferenced with respect to its direct neighbor, i.e., bipolar montage, to achieve high local specificity by removing effects of distant sources that spread equally to adjacent sites through volume conduction. All the data were epoched from –800 to 1,000 ms relative to the presentation of the first frame.

### Acquisition and preprocessing of the fMRI data

A Siemens 3T Trio system with a standard head coil at Beijing MRI Center for Brain Research was utilized to obtain T2*-weighted echo-planar images (EPIs) with blood oxygenation level-dependent contrast. The matrix size was 64 × 64 mm^3^, and the voxel size was 3.4 × 3.4 × 3 mm^3^. Thirty-six transversal slices of 3-mm thickness that covered the whole brain were acquired sequentially with a 0.75-mm gap (TR = 2.2 s, TE = 30 ms, FOV = 220 mm, flip angle = 90°). There was a single run of functional scanning, including 524 EPI volumes. The first five volumes were discarded to allow for T1 equilibration effects.

Data were preprocessed with Statistical Parametric Mapping software SPM12 (Wellcome Department of Imaging Neuroscience, London, UK; http://www.fil.ion.ucl.ac.uk). Images were realigned to the first volume to correct for interscan head movements. The mean EPI of each participant was then computed and spatially normalized to the MNI single-participant template using the “unified segmentation” function in SPM12. This algorithm is built on a probabilistic framework that enables image registration, tissue classification, and bias correction to be combined within the same generative model. The resulting parameters of a discrete cosine transform, which define the deformation field necessary to move individual data into the space of the MNI tissue probability maps, were then combined with the deformation field transforming between the latter and the MNI single participant template. The ensuing deformation was subsequently applied to individual EPI volumes. All images were thus transformed into standard MNI space and resampled to 2 × 2 × 2 mm^3^ voxel size. The data were then smoothed with a Gaussian kernel of 8-mm full-width half-maximum to accommodate interparticipant anatomical variability. Data were high-pass filtered at 1/128 Hz and analyzed with a general linear model (GLM) as implemented in SPM12. Temporal autocorrelation was modeled using an AR (1) process.

### Analysis of the behavioral data

For the behavioral data in the EEG, intracranial, and fMRI experiment, omissions and trials with RTs 3 standard deviations (SDs) away from the mean RT in each condition were first excluded from further analysis. For the calculation of accuracy rates in the two explicit conditions, the explicit trials at the short IFI with a judgment of GM and the explicit trials at the long IFI with a judgment of EM were considered as incorrect trials, which were discarded and excluded from further analysis. For both the EEG and fMRI experiment, paired *t* tests were performed to test the difference in the accuracy rates between the two types of explicit trials, the proportions of EM and GM trials in the bistable condition, and the mean RTs for the two explicit and the two bistable conditions, respectively.

### Alpha oscillation analysis of the EEG data

For all the electrodes in all the participants, a power spectrum (from 5 Hz to 30 Hz) was obtained through a Fast Fourier Transform (FFT) of all the trials (from –800 to 0 ms relative to the presentation of the first frame). An amplitude topographic map of the most prominent frequency band in the power spectrum was obtained. For each participant, the individual peak alpha frequency was determined as the value corresponding to the maximum peak frequency from the 800 ms of data prior to the presentation of the first frame within the 8–13 Hz range for the selected posterior electrodes. The Pearson product–moment correlation between the individual alpha frequency and the individual IFI threshold obtained from the psychophysical procedures was then calculated.

Instantaneous PAF and alpha power were analyzed using the methods and code developed by Cohen [58]. We chose only one electrode, which showed the strongest alpha amplitude among all the occipital electrodes in the posterior ROI for each participant, to calculate the PAF and the alpha power [51]. To avoid contaminations by the poststimulus signals, only the prestimulus period (from –800 to 0 ms) of the EEG signals were extracted, and all the poststimulus period signals (starting from 0 ms) were excluded. Furthermore, to avoid edge artifacts at the stimulus onset due to filtering, the prestimulus signals of each bistable trial were copied, flipped from left to right, and appended to the right side of the original data. These epochs were filtered between 8 and 13 Hz with a zero-phase, plateau-shaped bandpass filter with 15% transition zones. Phase angle and amplitude time series were extracted from the filtered data with a Hilbert transform. The alpha power was obtained by calculating the square of the amplitude. For the frequency calculation, the temporal derivative of the phase angle time series describes how phase changes over time and thus corresponds to the instantaneous frequency in Hz (when scaled by the sampling rate and 2*π*). Since noises in the phase angle time series can cause sharp, nonphysiological responses in the derivative, the instantaneous frequency was filtered with a median filter with an order of 10 and a maximum window size of 400 ms: data were median filtered ten times with 10 time windows ranging from 10 to 400 ms prior to averaging across trials. Since this analysis considers changes only in the instantaneous phase of the data, it is mathematically independent from the amplitude of the oscillation, except where amplitude is equal to zero and phase is undefined. Subsequently, the instantaneous PAFs were averaged across bistable EM and GM trials, respectively.

### Decoding analysis of the EEG data

Multivariate decoding techniques were further adopted to investigate how the PAF affects the representation contents of the bistable EM and GM percepts with the progress of time. For each participant, we first calculated the instantaneous alpha frequency for each time point in the prestimulus window (from –800 ms to 0 relative to the onset of frame 2) of each bistable trial, based on the one chosen electrode with the maximal alpha amplitude. Subsequently, statistical tests (paired *t* test) between the bistable EM and bistable GM conditions were performed at the group level. The significant time points were further selected as the time points of interest, and the PAF for each trial was determined by averaging the instantaneous alpha frequency across these significant time points (–570 to –350 ms relative to the presentation of frame 2; see S3 Fig). Subsequently, amplitude data epochs of all the bistable trials (–400 to 400 ms relative to the presentation of frame 2), right after the preprocessing steps and without any further processing steps (no spectral analysis applied), were sorted according to the calculated PAF of each trial and half split into the high PAF and the low PAF trial sessions. The bistable GM trials in the high PAF session and the bistable EM trials in the low PAF session were selected as the two types of trials in the efficient inference condition; the bistable EM trials in the high PAF session and the bistable GM trials in the low PAF session were selected as the two types of trials in the inefficient inference condition. Please note, the PAF of each bistable trial was used only as an indicator to categorize the bistable trials into the efficient versus inefficient conditions in a post hoc way but was never used as the actual data fed into the subsequent decoding analysis. To exclude potential confounds caused by different number of trials upon comparing different conditions, we matched the trial count in the above four types of trials by randomly selecting a subsample of trials from the conditions with more trials. We then applied a multivariate linear discriminant analysis to characterize the temporal dynamics that discriminated between the subjectively perceived bistable EM versus GM percepts for the efficient and inefficient inference condition, respectively.

Classifications were based on the regularized linear discriminant analysis to identify a projection in the multidimensional EEG data, *x*, that maximally discriminated between the two representations across all stimulus levels. Each projection is defined by a weight vector, *w*, which describes a one-dimensional projection *y* of the EEG data $y=\sum_{i}w_{i}x_{i}+c,$ with *i* summing over all channels and *c* a constant. The regularization parameter was optimized in preliminary tests and kept fixed for all the analyses. The decoding analysis was performed in a time-resolved manner by applying it to each time point sequentially, resulting in an array of classifiers, for example, *w*(t1), *w*(t2), *w*(t3) and so on. To improve the signal-to-noise ratio, the data were first averaged within a time window of 50 ms centered around the time point of interest. This process could introduce some contaminations from the poststimulus signals to the prestimulus signals around the stimuli onset: the signal at time 0 contains the information within –25 to 25 ms. However, the abovementioned contaminations can influence the prestimulus signals about 25 ms at most. Subsequently, the classifier performance was assessed not only at the time point used for training (for example, classifier *w*(t1) was tested at t1, *w*(t2) was tested at t2, and so on) but also on data from all the other time points (for example, classifier *w*(t1) was tested on all the time points t1, t2, t3, and so on). The performance of the classifier was quantified using the receiver operator characteristic (ROC), based on leave-one-out cross-validation within each participant. The above procedure resulted in a (training time) × (decoding time) temporal generalization matrix per condition.

### Alpha frequency analysis of the intracranial data

We first extracted the averaged alpha amplitude during the prestimulus period (–800 to 0 ms relative to the first frame) for each contact in the same manner as for the EEG analysis (using FFT). The first 10 contacts with the highest alpha amplitude (8–13 Hz) were then selected as ROIs for each patient. Subsequently, we adopted similar methods and procedures as in the EEG analysis to calculate the prestimulus instantaneous frequency for the bistable EM and GM trials for each contact, which was subsequently averaged across the 10 contacts.

### Statistical testing of the neurophysiology data

The difference between two conditions was statistically tested using nonparametric cluster-based permutation tests, which were implemented in customized scripts in MATLAB (The MathWorks). Specifically speaking, paired *t* tests were first calculated between the two conditions, for example, the temporal generalization matrices for the efficient versus inefficient inference conditions. Elements that passed a threshold value corresponding to a *p*-value of 0.05 were marked, and neighboring marked elements were identified as clusters. Cluster-based correction was applied when multiple time points were tested (Figs 3D, 4 and 5D–5G): data were first randomly shuffled 1,000 times (500 times in the decoding analysis); for each shuffle, the count of suprathreshold samples within a cluster was used to define the cluster size; and the largest cluster size was entered into a distribution of cluster sizes [106], which was expected under the null hypothesis. Clusters in the real data were considered as statistically significant only if they exceeded the size of 95th percentile of the null distribution of clusters, at α = 0.05.

### Statistical analysis of the fMRI data

At the individual level, the GLM was used to construct a multiple regression design matrix. The four experimental conditions were modeled as regressors of interest: explicit EM, explicit GM, bistable EM, and bistable GM. The four types of event were time locked to the onset of the first frame in each trial by a canonical synthetic hemodynamic response function and its first-order time derivative with an event duration of 0 s. In addition, all the omission trials and the outlier trials in which RTs were outside of the mean RT ± 3 SD were modeled separately as another regressor. The six head movement parameters derived from the realignment procedure were also included as confounds. Parameter estimates were subsequently calculated for each voxel using weighted least-square analysis to provide maximum likelihood estimators based on the temporal autocorrelation of the data. No global scaling was applied.

For each participant, simple main effects for each of the four experimental conditions were computed by applying appropriate “1 0” baseline contrasts, that is, experimental conditions versus implicit baseline (null trials). The four first-level individual contrast images were then fed into a within-participants ANOVA at the second group level employing a random-effects model (flexible factorial design in SPM12 including an additional factor modeling the subject means). In the modeling of variance components, we allowed for violations of sphericity by modeling nonindependence across parameter estimates from the same subject and allowing unequal variances between both conditions and participants using the standard implementation in SPM12. We were particularly interested in the differential neural activity between the two types of bistable trials (bistable EM versus bistable GM). Areas of activation were identified as significant only if they passed a conservative threshold of *p* < 0.005, family-wise error (FWE) corrected for multiple comparisons at the cluster level, with an underlying voxel level of *p* < 0.005, uncorrected [107].

### Statistical analysis on prestimulus neural activity of the fMRI data

To investigate how the prestimulus neural activity predicted the outcome of bistable perceptual grouping, a new GLM model was estimated. Given that the ITI was jittered between 2,000–3,000 ms and one-third of all the trials were null trials, the prestimulus periods of all the experimental trials were long enough and adequately jittered for the present statistical analysis on prestimulus neural activity. In the new GLM model, four types of new events were time locked to the time points after the participants made their responses in the preceding trials (“Trials N-1”) of the four types of experimental trials, i.e., the prestimulus preparation period of the current trial (“Trials N”). All the outliers, errors, and missed trials and trials preceded by outliers and errors were separately modeled as another regressor. In this way, parameter estimates in each of the four newly defined critical neural events indicate the height of prestimulus preparation neural activity prior to the actual presentation of the explicit EM, the explicit GM, the bistable EM, and the bistable GM stimuli. Brain regions of activation were identified as significant only if they passed a conservative threshold of *p* < 0.005 FWE correction for multiple comparisons at the cluster level, with an underlying voxel level of *p* < 0.005, uncorrected.

### PPI analysis on prestimulus neural activity of the fMRI data

Since the left IPS exhibited specific selectivity towards the bistable EM percepts during both the pre- (Fig 6C) and the poststimulus (Fig 6B) period, we used the left IPS as the seed region to perform the PPI analysis, focusing on the prestimulus period. For the PPI analysis, prestimulus neural activity (time locked to the responses in “Trials N-1”) in the left IPS was used as the physiological factor and the contrast of “bistable EM versus bistable GM” as the psychological factor. For each participant, the neural contrast of “bistable EM versus bistable GM” was first calculated in the individual level GLM. Subsequently, each participant’s individual peak voxel in the left IPS was determined as the maximally activated voxel within a sphere of 16-mm radius (i.e., twice smoothing kernel) around the coordinates of the peak voxel from the second-level group analysis (Fig 6B). Individual peak voxels from every participant are located in the same anatomical structure (left IPS MNI coordinates: *x* = –33 ± 6, *y* = –37 ± 7, *z* = 42 ± 6). Next, the left IPS time series in every participant were extracted from a sphere of 4-mm radius around the individual peak voxels. The PPI term was created for each participant by multiplying the deconvolved and mean-corrected BOLD signal in the given ROI (i.e., the physiological variable) with the psychological variable of interest (i.e., “bistable EM versus bistable GM”). After convolution with the HRF, mean correction, and orthogonalization, three regressors (the PPI term, the physiological variable, and the psychological variable) were entered into the GLM to reveal areas in which neural activations were predicted by the PPI term, with the physiological and the psychological regressors being treated as confounding variables. The PPI analysis was first carried out for each participant and then entered into a random-effects group analysis. Statistical significance was set to *p* < 0.005, uncorrected at the voxel level, with the cluster extent exceeding 100 voxels.

## Supporting information

S1 Table Patient information and behavioral performance in the intracranial experiment. Following information is reported for each patient: the EZ identified by the clinical investigation; the number of ELs; the total number of CHs. Task performance: ARs of the explicit EM and GM condition; reported rates of GM in the bistable condition. AR, accuracy rate; CH, electrode contact; EL, implanted electrode shaft; EM, element motion; EZ, epileptogenic zone; GM, group motion.
(DOCX)

S2 Table Brain activations in the main Fig 6B and 6C. Brain regions showing significant relative increases of BOLD response associated with the bistable EM and bistable GM trials before and after the presentation of the first frame. BOLD, blood-oxygen–level dependent; EM, element motion; GM, group motion.
(DOCX)

S3 Table Brain activations in the main Fig 6D. Brain regions that showed higher prestimulus functional connectivity with the left IPS (–32, –38, 38) in the bistable EM than bistable GM trials. EM, element motion; GM, group motion; IPS, intraparietal sulcus.
(DOCX)

S1 Video Demo of explicit EM. EM, element motion.
(MP4)

S2 Video Demo of explicit GM. GM, group motion.
(MP4)

S1 Fig Psychometric fitting results for each participant in fMRI experiment. Underlying data available at https://osf.io/tze94/. fMRI, functional magnetic resonance imaging.
(TIF)

S2 Fig Correlation between the occipital alpha cycle in each individual subject and the individual transition IFI threshold. Dashed lines indicate 95% confidence intervals around the linear fit line. Underlying data available at https://osf.io/tze94/. IFI, interframe interval.
(TIF)

S3 Fig Instantaneous PAF relative to the representation of the second frame revealed higher alpha frequency preceding the bistable GM trials than the bistable EM trials. Significant time points are indicated by the horizontal black bar (cluster-based correction, *p* < 0.05). Shaded regions denote ±1 within-subjects SEM. Underlying data available at https://osf.io/tze94/. EM, element motion; GM, group motion; PAF, prestimulus alpha frequency; SEM, standard error of the mean.
(TIF)

## Abbreviations

AUC: area under the receiver operator characteristic
BOLD: blood-oxygen–level dependent
DMN: default-mode network
EEG: electroencephalography
EM: element motion
EPI: echo-planar image
FFT: Fast Fourier Transform
fMRI: functional magnetic resonance imaging
FWE: family-wise error
GLM: general linear model
GM: group motion
IFG: inferior frontal gyrus
IFI: interframe interval
IPS: intraparietal sulcus
LOC: lateral occipital cortex
MPFC: medial prefrontal cortex
MT: middle temporal gyrus
PAF: prestimulus alpha frequency
PPI: psychophysiological interaction
ROC: receiver operator characteristic
RT: reaction time
SD: standard deviation
SEM: standard error of the mean
SMA: supplementary motor area
TR: repetition time
2AFC: two-alternative forced choice
