# Supplementary material for: Perceptual inference employs intrinsic alpha frequency to resolve perceptual ambiguity
Source: PLoS Biol. 2019 Mar 13;17(3):e3000025. doi: 10.1371/journal.pbio.3000025 (PMC6433295; doi:10.1371/journal.pbio.3000025)
Supplement: S1 Table — Following information is reported for each patient: the EZ identified by the clinical investigation; the number of ELs; the total number of CHs. Task performance: ARs of the explicit EM and GM condition; reported rates of GM in the bistable condition. AR, accuracy rate; CH, electrode contact; EL, implanted electrode shaft; EM, element motion; EZ, epileptogenic zone; GM, group motion. (DOCX) [file pbio.3000025.s001.docx]

**Patient information and behavioral performance in the intracranial experiment.**

| **ID** | **Sex** | **Age** | **EZ** | **#EL** | **#CH** | **IFI**  **Threshold** | **Task Performance** | | |
| --- | --- | --- | --- | --- | --- | --- | --- | --- | --- |
|  |  |  |  |  |  |  | **Explicit EM (AR)** | **Explicit GM (AR)** | **Bistable GM (Rates)** |
| 1 | M | 22 | Right parietal | 13 | 185 | 146 ms | 89% | 89% | 47% |
| 2 | M | 36 | Left temporal | 10 | 148 | 90 ms | 100% | 100% | 50% |
| 3 | F | 23 | Left posterior temporal | 11 | 147 | 116 ms | 82% | 96% | 36% |
| 4 | F | 16 | Left medial temporal | 11 | 145 | 124 ms | 94% | 98% | 48% |
