# Supplementary material for: Perceptual inference employs intrinsic alpha frequency to resolve perceptual ambiguity
Source: PLoS Biol. 2019 Mar 13;17(3):e3000025. doi: 10.1371/journal.pbio.3000025 (PMC6433295; doi:10.1371/journal.pbio.3000025)
Supplement: S2 Table — Brain regions showing significant relative increases of BOLD response associated with the bistable EM and bistable GM trials before and after the presentation of the first frame. BOLD, blood-oxygen–level dependent; EM, element motion; GM, group motion. (DOCX) [file pbio.3000025.s002.docx]

**Brain activations in the main Figure 6A, B.**

| **Anatomical Region** | **Hemisphere** | **Cluster Peak (mm)** | ***t*-Score** | ***k*_E_ (voxels)** |
| --- | --- | --- | --- | --- |
| **Peri-stimulus Neural Activity** | | | | |
| **a. Bistable_EM > Bistable_GM** | |  |  |  |
| Inferior parietal gyrus | L | -32, -38, 38 | 4.80 | 1413 |
| *Superior parietal gyrus* | *L* | *-22, -58, 48* | *4.5* |  |
| **b. Bistable_GM > Bistable_EM** | |  |  |  |
| Superior medial prefrontal gyrus | L | -4, 46, 50 | 5.9 | 1165 |
|  |  |  |  |  |
| **Pre-stimulus Neural Activity** | | | | |
| **c. Bistable_EM > Bistable_GM** | |  |  |  |
| Inferior parietal gyrus | L | -28, -48, 46 | 5.04 | 824 |
| *Superior parietal gyrus* | *L* | *-20, -70, 46* | *4.08* |  |
| Inferior frontal gyrus | R | 48, 16, 20 | 4.48 | 631 |
| Inferior frontal gyrus | L | -50, 36, 12 | 4.31 | 608 |

The coordinates (x, y, z) correspond to MNI coordinates. Displayed are the coordinates of the maximally activated voxel within a significant cluster as well as the coordinates of relevant local maxima within the cluster (*in Italics*).
