# Supplementary material for: Perceptual inference employs intrinsic alpha frequency to resolve perceptual ambiguity
Source: PLoS Biol. 2019 Mar 13;17(3):e3000025. doi: 10.1371/journal.pbio.3000025 (PMC6433295; doi:10.1371/journal.pbio.3000025)
Supplement: S3 Table — Brain regions that showed higher prestimulus functional connectivity with the left IPS (–32, –38, 38) in the bistable EM than bistable GM trials. EM, element motion; GM, group motion; IPS, intraparietal sulcus. (DOCX) [file pbio.3000025.s003.docx]

**Brain activations in the main Figure 6C.**

| **Anatomical Region** | **Hemisphere** | **Cluster Peak (mm)** | ***t*-Score** | ***k*_E_ (voxels)** |
| --- | --- | --- | --- | --- |
| SMA | R | 6, 14, 54 | 4.62 | 616 |
| Inferior frontal gyrus | L | -34, 14, -8 | 4.26 | *252* |
| Inferior parietal gyrus | R | 48, -36, 58 | 3.98 | 146 |
| Inferior frontal gyrus | R | 36, 18, -2 | 3.95 | 137 |

The coordinates (x, y, z) correspond to MNI coordinates. Displayed are the coordinates of the maximally activated voxel within a significant cluster.
