# Supplementary figures and images for: Perceptual inference employs intrinsic alpha frequency to resolve perceptual ambiguity
Source: PLoS Biol. 2019 Mar 13;17(3):e3000025. doi: 10.1371/journal.pbio.3000025 (PMC6433295; doi:10.1371/journal.pbio.3000025)

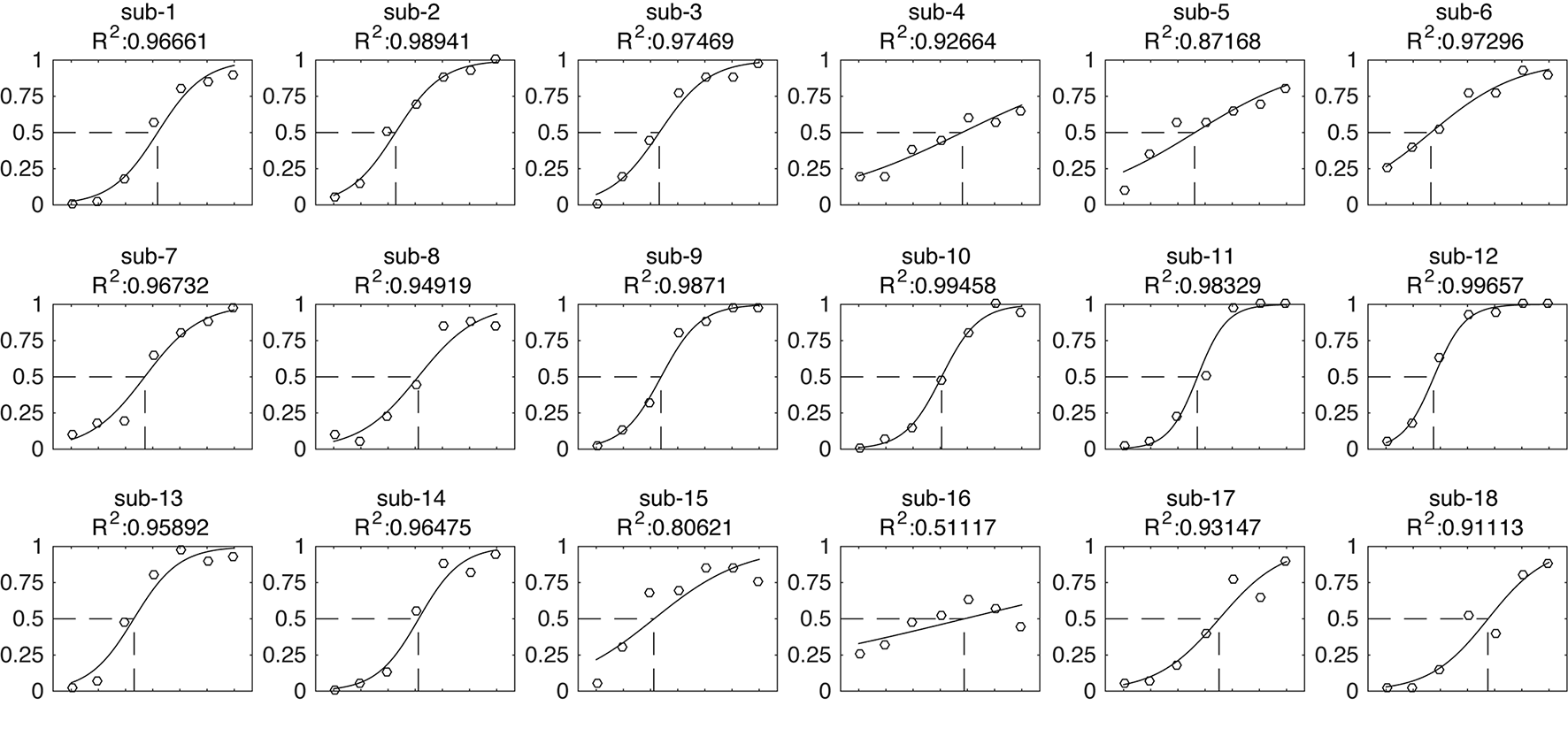

Supplement: S1 Fig — Underlying data available at https://osf.io/tze94/. fMRI, functional magnetic resonance imaging. (TIF) [file pbio.3000025.s006.tif]

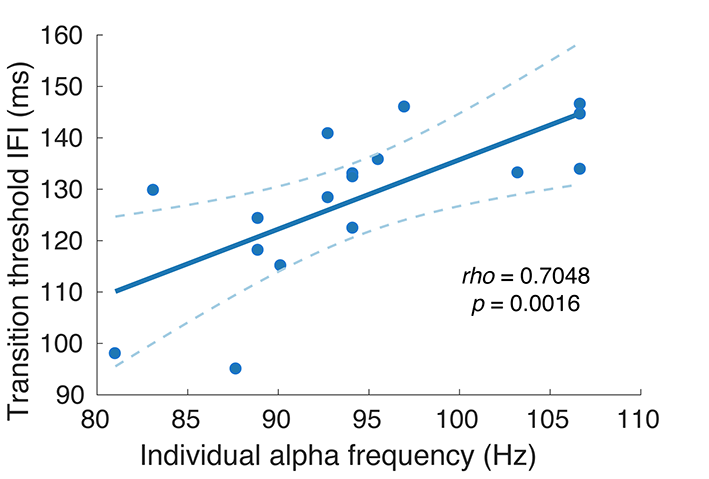

Supplement: S2 Fig — Dashed lines indicate 95% confidence intervals around the linear fit line. Underlying data available at https://osf.io/tze94/. IFI, interframe interval. (TIF) [file pbio.3000025.s007.tif]

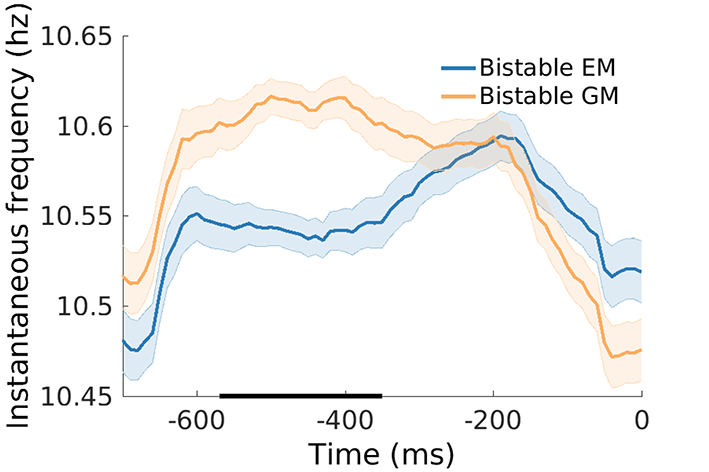

Supplement: S3 Fig — Significant time points are indicated by the horizontal black bar (cluster-based correction, p < 0.05). Shaded regions denote ±1 within-subjects SEM. Underlying data available at https://osf.io/tze94/. EM, element motion; GM, group motion; PAF, prestimulus alpha frequency; SEM, standard error of the mean. (TIF) [file pbio.3000025.s008.tif]
